# A computational framework for boosting confidence in high-throughput protein-protein interaction datasets

**DOI:** 10.1186/gb-2012-13-8-r76

**Published:** 2012-08-31

**Authors:** Raghavendra Hosur, Jian Peng, Arunachalam Vinayagam, Ulrich Stelzl, Jinbo Xu, Norbert Perrimon, Jadwiga Bienkowska, Bonnie Berger

**Affiliations:** 1 Computer Science and Artificial Intelligence Laboratory, 32 Vassar Street, MIT, Cambridge, MA 02139, USA; 2 Toyota Technological Institute, 6045 S. Kenwood Ave, Chicago, IL 60637, USA; 3 Department of Genetics, 77 Avenue Louis Pasteur, Harvard Medical School, Boston, MA 02115, USA; 4 Otto-Warburg Laboratory, Ihnestraβe 63-73, Max Planck Institute for Molecular Genetics, Berlin D14195, Germany; 5 Howard Hughes Medical Institute, 20 Shattuck Street, Boston, MA 02115, USA; 6 Computational Biology group, Biogen Idec, 14 Cambridge Center, Cambridge, MA 02142, USA; 7 Department of Mathematics, 77 Massachusetts Avenue, MIT, Cambridge, MA 02139, USA

## Abstract

Improving the quality and coverage of the protein interactome is of tantamount importance for biomedical research, particularly given the various sources of uncertainty in high-throughput techniques. We introduce a structure-based framework, Coev2Net, for computing a single confidence score that addresses both false-positive and false-negative rates. Coev2Net is easily applied to thousands of binary protein interactions and has superior predictive performance over existing methods. We experimentally validate selected high-confidence predictions in the human MAPK network and show that predicted interfaces are enriched for cancer -related or damaging SNPs. Coev2Net can be downloaded at http://struct2net.csail.mit.edu.

## Background

Protein-protein interactions (PPIs) play a critical role in all cellular processes, ranging from cellular division to apoptosis. Elucidating and analyzing PPIs is thus essential to understanding the underlying mechanisms in biology. Indeed, this has been a major focus of research in recent years, providing a wealth of experimental data about protein associations [1-9]. Current PPI networks have been constructed using a number of techniques, such as yeast-two-hybrid (Y2H), co-immunopurification or coaffinity purification, followed by mass spectroscopy and curation of published low-throughput experiments [10-16]. Despite this tremendous push, the current coverage of PPIs is still rather poor (for example, < 10% of interactions in humans) [17]. Additionally, despite considerable improvements in high-throughput (HTP) techniques, they are still prone to spurious errors and systematic biases, yielding a significant number of false-positives and false-negatives [18-21]. This limitation impedes our ability to assess the true quality and coverage of the 'interactome' [22-24].

Akin to sequencing of the human genome, complete high-confidence descriptions of PPIs is a fundamental step towards human interactome mapping [22,25]. Also present are the challenging issues of data quality and size estimation, as encountered in the human genome project [23,24,26]. However, unlike the challenges faced previously with sequencing, we still do not understand the rules of association of protein molecules, and are unable to distinguish between biophysical interactions, true biological interactions and false-positives [20]. Further unresolved questions as to the proportion of experimental artifacts in the current interactomes are coming to light as a consequence of the low degree of overlap between data curated from multiple HTP (as well as low-throughput) studies [27].

Several attempts have been made to characterize the quality of the interactions obtained from HTP experiments [7,23,24,28-31]. Experimental methods aim to limit false discovery by performing multiple iterations of the screen, which are time-consuming and expensive [29]. Secondary data, such as co-expression, co-localization, ontology correlation, topological features and orthology information are often used to further improve confidence in predicted interactions [32,33]. In addition to non-trivial correlations between these features (that is, co-expression need not imply interaction), these data are not complete for all proteins. Furthermore, as more and more genomes are sequenced, only a fraction of proteins will have additional data to complement any experimental HTP study. Techniques developed from integrating interactions observed in common across multiple secondary experimental assays of an initial network are laborious, expensive and time-consuming. Moreover, as suggested by Venkatesan *et al. *[22] and Cusick *et al. *[27], the low overlaps achieved across different datasets highlight the differences in sampling and biases in experimental techniques rather than pinpoint the true interactions. Further, in many experimental methods, the confidence of observations is evaluated for that specific technique - they are seldom generalizable. Thus, cost-effective and high-confident strategies are clearly required to complete the human interactome.

Recently, a number of algorithms have been developed to predict protein interactions by integrating complementary data such as sequence features and structural features [12,34-42]. Also recently, computational approaches to PPI prediction using structural information have been gaining much attention due to the rapid growth of the Protein Data Bank (PDB) [32,35,43-65]. An important advantage of structure-based approaches is their ability to identify the putative interface, thereby providing more information than any other HTP method. The common strategy of structure-based methods is to find a best-fit template complex structure for the two query sequences; the prediction is then based on the similarity of the two proteins to the template complex. Threading-based approaches extend coverage further 'into the twilight zone', making accurate predictions even when there is low sequence similarity (typically < 40%) between the query proteins and the best-fit template complex [32,49,66]. However, to the best of our knowledge, there have been no studies that integrate HTP techniques with PPI prediction algorithms to quantitatively address both false-negative and false-positive issues.

In this paper, we introduce a general framework to predict, assess and boost confidence in individual interactions inferred from a HTP experiment. Our contribution is three-fold: 1) we develop a novel computational algorithm to quantitatively predict interactions, given just the protein sequences; 2) we show how the algorithm can be used in a general framework to quantify confidence in observed interactions; and 3) we demonstrate the utility of our structure-based framework in providing biologically significant additional information about binding sites, which is not provided by any other HTP method (either computational or experimental). We first validate our method on a high-confidence network in the recently investigated human mitogen-activated protein kinase (MAPK) interactome [67,68]. We experimentally validate predicted high-confidence interactions for the MAPK interactome using a complementary assay and show that the concordance between prediction and experimental validation is as good as the overlaps achieved in previous protocols involving multiple secondary assays [25]. Finally, we show that the interfaces predicted by our algorithm are enriched for functionally important sites in the context of signaling networks; and utilize this information to hypothesize a novel regulatory mechanism involving crosstalk between the insulin and stress-response pathways via interactions between MAPK6, YWHAZ and FOXO3 proteins.

## Results

### The Coev2Net framework for quantifying confidence in protein interactions

**Figure 1:**
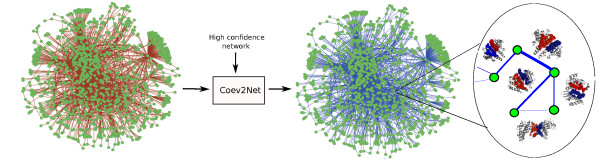
**Framework for assessing confidence in a HTP PPI screen**. Coev2Net, trained on a high-quality PPI network, is able to assign structure-based confidence scores for HTP PPI networks. Each node represents a protein and each edge the putative interaction between the two proteins. The thickness of an edge describes structure-based confidences of putative PPIs.

We developed Coev2Net (Figure 1), a framework for assessing confidence in protein interactions. To quantify confidence in an interactome, we incorporate high-confidence data sources, namely low-throughput interactions and structural information. The framework gives a confidence score for each interaction, along with a predicted model of the binding interface for the proteins (Figure 1).

**Figure 2:**
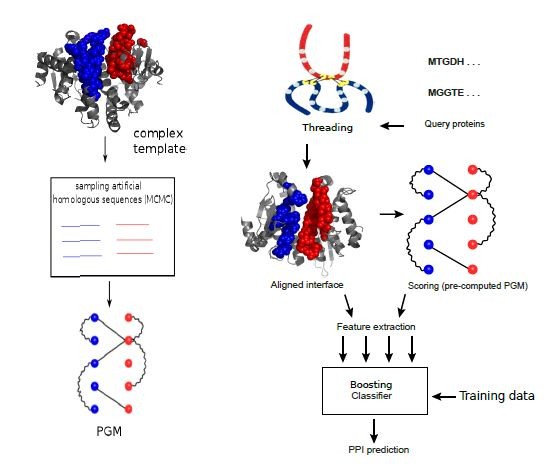
**Flowchart of Coev2Net**. Left: Markov chain Monte Carlo (MCMC) sampling to generate synthetic homologous sequences for each complex template. Right: 1) for given query protein pairs, the best template (from the structural library) is identified by protein threading; 2) structural and sequence features are extracted from the interfacial alignment and residue correlations scored with respect to the profile PGM; and 3) a classifier gives the probability of interaction for the query protein pair.

Inputs to the framework are a high-confidence network (usually much smaller than the HTP screen) and the interactions identified from the HTP experiment for which one wishes to quantify confidence. For every pair of interaction in the HTP screen, Coev2Net provides a score to assess their likelihood of being co-evolved from interacting homologous sequences (see Materials and methods). To do this, Coev2Net first predicts a likely interface model for the two proteins, by threading [69] the sequences onto the best-fit template complex in our library. It then computes the likelihood of co-evolution of the two proteins (that is, of the predicted interface) with respect to a probabilistic graphical model (PGM) induced by the aligned interfaces of artificial homologous sequences (Figure 2; Materials and methods; Additional file 1). By generating artificial sequences, we enrich the interfacial sequence/structure profiles for those protein-pairs with sparse interacting-sequence profiles and thus improve protein interface scoring accuracy. Note that this enrichment is carried out for all protein pairs, irrespective of the information content in their individual sequence profiles. These PGM scores are then input into a classifier trained on a small high-confidence network to compute a score between 0 and 1, representing the confidence of our method in that interaction (Figure 1). High-scoring interactions can then be investigated further using a secondary experimental assay or taken as true positives for subsequent analyses. Additionally, since Coev2Net is a structure-based algorithm, it also produces as output a putative interface for the interacting pair (Figure 2). This information can be analyzed to design site-directed experiments to further characterize the specificity of the interaction.

### Benchmarking Coev2Net

#### SCOPPI

We first benchmark Coev2Net on SCOPPI [70], a protein complex database. The database is divided into interacting family pairs for which multiple complexes have been solved. Rigorous cross-validation tests on the database indicate that Coev2Net achieves high accuracies, thereby validating our approach of modeling interface co-evolution as a high-dimensional sampling problem (Figure S3 in Additional file 1). For the cross-validation tests, we considered only those family pairs in SCOPPI that have at least three non-redundant (sequence id < 50%) complexes. We randomly selected one as the test complex and used the other complexes within our Coev2Net protocol to simulate interacting homologs and construct the PGM (Figure 2). We additionally compared Coev2Net's performance on the SCOPPI dataset to another structure-based method, PRISM [45]. PRISM first identifies similar templates to two query structures by structural alignment. The final prediction is based upon the energy of complex formation calculated by docking these two predicted interfaces. We find that Coev2Net's performance, measured in terms of sensitivity and specificity, is much better than PRISM's on this dataset (Figure S3 in Additional file 1).

Furthermore, Coev2Net also performs well on SCOPPI family pairs not having more than two non-redundant complexes, indicating Coev2Net's ability to deal with limitations of both structural and sequence training data (Figure S3 in Additional file 1).

### MAPK interactome validation

**Figure 3:**
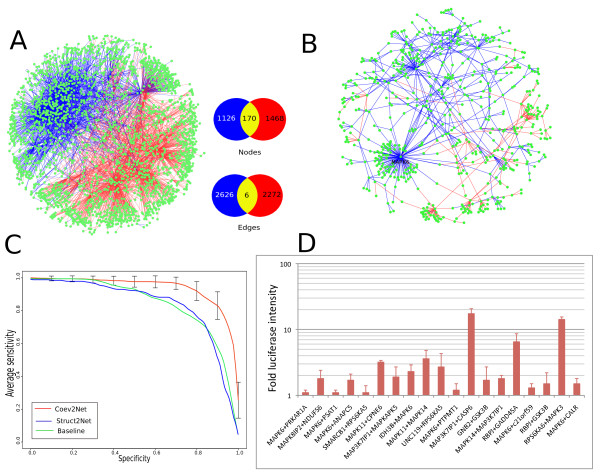
**MAPK interactome analysis and validation**. **(a) **Overlap of the Vinayagam (blue) and Bandyopadhyay (red) datasets (left). The study by Bandyopadhyay *et al. *reveals 2,269 interactions with 641 'core' interactions supported by multiple lines of evidence, whereas the Vinayagam dataset has 2,626 interactions connecting 1,126 proteins. Differences in the two experimental techniques are highlighted by the fact that only 170 nodes and 6 interactions overlap in the two sets. **(b) **Coev2Net predicted high-confidence network is shown on the right. Edge colors correspond to the dataset they come from. MAPK6 has the highest degree, and its label is shown explicitly. **(c) **Comparisons of performance on MAPK network for Coev2Net and Struct2Net (iWRAP+DBLRAP) [32,49,66] in terms of sensitivity and specificity. Coev2Net performs much better than previous methods on this dataset (core network of Bandyopadhyay *et al.*), and its performance is robust with respect to the randomness in MCMC sampling. The classifier (Figure 2) is trained and tested via five-fold cross-validation on the core network. The MCMC procedure is repeated five times to assess robustness of the predictions and the corresponding error bars are indicated. 'Baseline' method represents a logistic regression classifier with just the alignment features and no PPI (inter-protein) features. **(d) **Experimental validation of predicted high-confidence interactions using LUMIER assay. Typically a fold increase of 1.5 is considered as a true positive.

To test the framework's ability to predict interactions for which there is often no structural data available and to assign confidence values to interactions, we re-trained Coev2Net on a high-quality human MAPK PPI network [67] and tested it on another high-quality MAPK network [68] (Figure 3a-c). Oddly, these two MAPK networks are almost disjoint with only 6 overlapping interactions out of 4,904 total interactions (Figure 3a). In the Bandyopadhyay set [67], we could make predictions for 461 interactions, in the Vinayagam set [68], 1,025 interactions, and in the negatome (PDB-negative set, see *Datasets *in Additional File 1), 330 non-interactors. To check for known complexes in the two MAPK networks, for each interaction, we ran BLAST against the entire PDB to identify homologous complexes. We were able to find only 22 pairs for which a solved homologous complex exists in the PDB (we used an E-value cutoff of 1e-40). On the other hand, our threading-based approach can make predictions for approximately 1,500 interactions in the MAPK networks, indicating that our method extends predictions to those pairs for which a clear homologous complex does not exist. The Bandyopadhyay set was further divided into a 'core' set of interactions (640), of which we could make predictions for 173 pairs. The definitions for core set and non-core set were taken as in the original citation [67]. This core set of interactions contains high-confidence interactions that are conserved in yeast [67].

To test the accuracy of Coev2Net's predictions, we first validated our method via five-fold cross-validation on the high-confidence core set of interactions in the Bandyopadhyay set (Figure 3c). In addition, to assess the contribution of co-evolutionary profiles for PPI predictions, we compared the performance of our method to Struct2Net and a 'baseline' classifier that is trained on just the threading-based features (no inter-protein features). Note that all methods are evaluated on the same dataset (the core set). Figure 3c clearly shows that Coev2Net accurately predicts interactions even when only a distant homologous complex is available and thus fills the existing gap in structure-based methods for PPI prediction. In addition, Figure 3c also shows that including long-distance correlations as in Coev2Net aids in PPI prediction as compared to other threading-based methods.

We trained our final classifier on the entire Bandyopadhyay core data set, and predicted interactions in the Vinayagam dataset. For the predictions made for the latter dataset, we found that the experimentally validated coverage of our method (approximately 55% with a confidence-score cutoff of 0.6) is significantly higher than that reported by other prediction methods based on conservation, genomic data, gene ontology annotation and literature extractions (approximately 14% to approximately 28%) [29], although each method was evaluated on a different network. Here, coverage is defined as the percentage of total predicted interactions for which we make a positive prediction and that were validated experimentally in the Y2H screen (571 predicted positive out of 1,025 in the Vinayagam dataset). The cutoff of 0.6 was chosen since it corresponds to the maximum specificity and sensitivity of the logistic-regression classifier on the Bandyopadhyay core dataset.

Moreover, our predicted confidence scores are highly correlated with the experimental observation frequencies of Y2H screens on this network (Vinayagam dataset). To assess significance, we divided our predictions into high confidence and low confidence based on the probability cutoff of 0.6. To categorize interactions as true positive or true negative in the Y2H screens, we assumed the cutoffs employed in Schwartz *et al. *(for a false discovery rate < 5%, true positive interactions should be observed at least twice when tested with < 5 independent assays, and at least three times when tested with more assays) [29]. We then populated a 2 × 2 contingency table to test for association between our predicted label (interacting or non-interacting) and experimentally predicted label. We find that the predicted interactions correlate (*P*-value < 0.01, Fisher's test) with those deemed likely true positives from an experimental standpoint. Encouragingly, the percentage of our framework's predicted true positive interactions that are confirmed positive (from an experimental standpoint) in the Vinayagam dataset is roughly 52% (294 true positive, 571 predicted positive, a two-fold increase compared to previous methods on Y2H retesting of computational predictions [29]. Alternatively, training Coev2Net on the high confidence network in the Vinayagam dataset and testing it on the Bandyopadhyay core network yields similar results. By predicting only a fraction of interactions with high confidence, Coev2Net enables us to focus on only the most likely interactions, enabling a more accurate understanding of the biology (Figure 3b).

### Experimental validation of predictions

The confidence scores given by our framework can be used to design additional experiments to enhance the quality of the initial interactome. We tested 19 randomly chosen high confidence interactions (confidence score > 0.6) using a complementary assay (LUMIER) [71]. Each pair, along with a control, was tested at least three times using the LUMIER assay. To confirm an interaction, the average result (that is, fold change in luciferase intensity [RLU] as measured in a TECAN Infinite M200 luminescence plate reader) across the repeats had to be greater than 1.5 times the control. Of the 19 interactions, 14 exhibited luciferase intensity greater than 1.5 times the control (Figure 3d). Additionally, if the repeat experiments were too variable to confidently assess the interaction (as measured using a z-score), the interaction pair was discarded. The z-score is calculated as:

$$z_{LUMIER}=\frac{\bar{RLU}-\bar{RLU_{control}}}{\sigma_{RLU}}$$

Eight out of the 19 interactions were discarded in this way as they registered a z-score of less than 1.5 and were deemed too variable. For additional experimental details we refer the readers to a more comprehensive interactome mapping analysis in [72]. Notably, 10 out of the remaining 11 were confirmed as true interactions, that is, registering average intensity above 1.5 times the control. Overlaps achieved by our method compare favorably with previous approaches, such as Braun *et al. *[25], in which an initial positive reference set was re-tested experimentally using a LUMIER assay (Table 1). Furthermore, we evaluated the sequence identities between the interacting sequences and the templates used for predicting their interaction (Table 1; Additional file 1. Interestingly, we find that all of them have a medium to low average sequence identity (15 to 30%), indicating that Coev2Net yields accurate predictions even in the 'twilight zone' of sequence identities, where traditional homology methods usually fail. For example, IBIS [73], another homology/structure-based method, can detect only two pairs from the ten detected by Coev2Net and experimentally validated by the LUMIER assay.

**Table 1:** Comparison of overlaps achieved by Braun *et al. *and our method when some of the initial Y2H interaction pairs are re-tested using LUMIER assay

Yeast strains implementation	Number validated (LUMIER)	Y2H PPIs	Percentage overlap
Y strain 2 m 1 reporter 1 mM_3-AT (Braun *et al.*)	19	33	57
Y strain 2 m 2 reporters 1 mM_3-AT (Braun *et al.*)	13	22	59
Y strain CEN 1 reporter 1 mM_3-AT (Braun *et al.*)	17	23	74
MaV CEN 2 reporters 20 mM_3-AT (Braun *et al.*)	9	14	64
Our prediction	**14**	**19**	**74**
Our prediction^a^	**10**	**11**	**91**

^a^These pairs have LUMIER assay z-scores > 1.5.

### Abundance of missense SNPs at predicted interfaces

**Figure 4:**
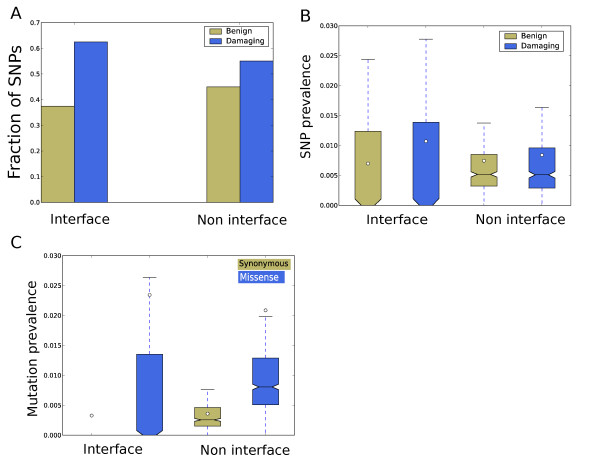
**Predicted interfaces are enriched for SNPs in the Coev2Net predicted high-confidence MAPK network**. **(a) **Relative distribution of PolyPhen annotated mutations at the interface and non-interface. **(b) **SNP (PolyPhen annotated) prevalence at the interface and non-interface. **(c) **Somatic mutations characterized as 'missense' preferentially fall on the interface (bottom). The white circles represent corresponding means. Error bars represent the 75% to 25% data range.

In addition to the confidence scores, Coev2Net also provides a putative interface for the interaction. These interfaces can yield novel mechanistic insights into the PPI and provide hypotheses about disease-associated mutations that occur at the interface. Missense SNPs occurring at the interface can potentially disrupt the interaction between the proteins, leading to abnormal functioning of the cell. We analyzed the predicted interfaces for existence of PolyPhen2 annotated missense mutations in dbSNP (build 131) [74]. PolyPhen2 classifies a SNP as 'benign', 'probably damaging', 'possibly damaging' or 'unknown' based on various features, including conservation score, monomeric structure score (when available) and physicochemical properties [75,76]. It does not, however, account for SNPs occurring in potential interacting regions. Interestingly, SNPs annotated as damaging by PolyPhen2 are preferentially observed at the interface compared to non-interfaces (*P *= 0.0075, Fisher's exact test; Figure 4a). Furthermore, if we take into account the number of interface and non-interface sites, we find that the predicted interfaces are enriched for damaging SNPs compared to the rest of the protein (P < 7e-8, Fisher exact test). The same analysis with SNPs classified as benign by PolyPhen2 does not show up as highly significant (*P *= 0.06). We further analyzed the distribution of the SNPs in terms of their density at the interface and non-interface. Here again, we find that damaging SNPs are preferentially located on the interface. We find that the average density of damaging SNPs at the predicted interfaces is significantly higher than their density at non-interface positions (Figure 4b; *P *< 1e-10, Mann-Whitney test), a bias also observed by Wang *et al. *recently [63]. For benign SNPs, the average density at the interface is lower than that at non-interfaces (Figure 4b; *P *< 1e-10, Mann-Whitney test). These analyses show that there is an evolutionary pressure to admit only benign SNPs at the interface, since any potentially damaging SNP will hinder the interaction.

To investigate the structural distribution of annotated mutations, we analyzed somatic mutations characterized in cancer to see if there is any preference for their location on the protein. We analyzed annotated mutations in the coding region deposited in the Cosmic database for their predicted location [77]. We only considered mutations that are annotated as either synonymous or missense. Interestingly, for these mutations we find that missense mutations are more prevalent, on average, at the PPI interface than synonymous mutations (*P *< 10e-20, Mann-Whitney test; Figure 4c). This suggests that these mutations might be responsible for disruption of PPIs and the aberrant molecular signaling associated with cancer.

**Figure 5:**
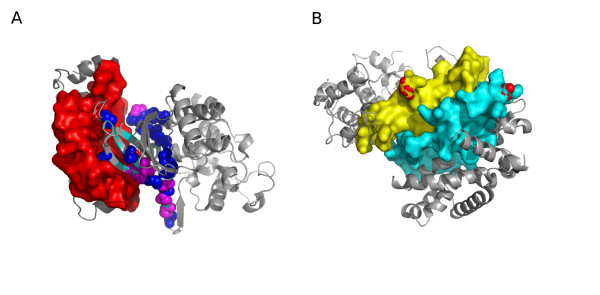
**Functional insights from predicted interface**. **(a) **Predicted interface for the interaction between BRAF (light blue) and PAK2 (red surface). Cancer-associated mutations that are annotated are shown in magenta. In dark blue we indicate mutations that are predicted to be associated with cancer but with no current annotations. The rest of the template structure is shown in gray. Mutations were taken from MoKCa database [78]. **(b) **Predicted interface for the interaction between MAPK6 (yellow) and YWHAZ (cyan). Phosphorylation sites on the proteins are indicated in red (S189 for MAPK6 and S184 for YWHAZ). The template used for the prediction was 1F5Q (chains A and B).

Finally, we looked at the predicted locations for some of the un-annotated mutations in kinases (from the MoKCa database [78]). As an example, we considered the BRAF protein as it contained the highest number of annotated mutations in the database. Coev2Net predicts an interaction between BRAF and PAK2, using the template structure 1G3N (chains E and F). Figure 5a shows the predicted interface for this interaction, with the annotated (magenta) and un-annotated (dark blue) mutations indicated. The presence of these mutations at the interface of the interacting proteins gives us an added insight into the investigation of such variations. Further study using this information can provide mechanistic details about how such mutations disrupt normal cellular signaling.

### Novel potential cross-talk regulatory mechanism

Phosphorylation sites have been observed to be enriched at interfaces in solved structures [79]. This observation has mechanistic implications as the PPI can be used as an additional regulatory mechanism for phosphorylation, or the interaction could be a precursor to phosphorylation. An example for such a mechanism is found in the signaling protein YWHAZ [80]. Its phosphorylation is regulated by its dimerization, which buries the phospho-sites on YWHAZ [81]. Our predictions revealed an interesting observation that suggests similar regulatory mechanisms in the MAPK interactome. Coev2Net predicts an interaction between MAPK6 and YWHAZ. Both are important signaling proteins, with much known about YWHAZ, including the experimental observation that MAPK8 regulates phosphorylation at S184 [82]. Relatively less is known about MAPK6's function and its substrates [83]. However, it is known that S189 is a phospho-site regulated by PAK1, PAK2 and PAK3 [84-86]. Interestingly, we found that the phosphorylation sites for both MAPK6 (S189) and YWHAZ (S184) lie within the predicted interface for the interaction (Figure 5b). This structural observation could imply that the interaction regulates downstream activities of MAPK6 and YWHAZ by controlling their phosphorylation. The most likely mechanism is that MAPK6 phosphorylates YWHAZ, thereby preventing its dimerization and regulating downstream activities of YWHAZ. Additionally, Coev2Net also predicts an interaction between MAPK6 and FOXO3. From a signaling context, these observations suggest a possible mechanism of crosstalk between the MAPK and insulin pathways. Analysis and validation of such a hypothesis is, however, beyond the scope of the present study.

## Discussion

We have proposed a novel structure-based computational approach to identify PPIs on a genome-wide scale. Using structural features, we have demonstrated that our method can not only identify true-interactions better than previous approaches, but also provide key biological insights that are absent from HTP experiments.

While it has been shown previously for some families that residues in and around the interface have correlated evolutionary histories, extracting such robust correlation signals for predictive purposes on a genome scale has remained difficult due to limited known interacting homologs. In the context of homology search for only monomers, enriching a multiple sequence alignment with artificial sequences has proven to be effective in the case of limited homologs [87,88]. Utilizing a statistical model for constructing evolutionarily correlated interacting homologs for a given interacting pair of proteins, we are able to simulate homologous sequences and predict PPIs from correlations at the interface of these homologs. The excellent performance of our method helps corroborate the hypothesis of residue-level correlations for a wide variety of PPIs and provides an efficient way of using these correlations for predictive purposes.

As more and more HTP data for mapping the interactome are gathered, there would be a necessary demand for automatic protocols to evaluate the data quality and estimate the confidence in individual interactions. In particular, transient interactions have been notoriously difficult to elucidate and validate. We have shown that confidence in PPIs investigated through HTP techniques can be quantified and enhanced by our proposed complementary structure-based PPI prediction algorithm. Our PPI predictions on recent HTP human MAPK interactomes and further experimental validations have indicated the efficacy of our predicted confidence scores. Moreover, since our framework requires only the sequences of the two candidate proteins, it can be used as a complementary feature to other methods that rely on additional features [31,89].

Limited studies have been undertaken to link structural features to genome-wide interactomes to gain a mechanistic understanding of underlying biological processes. Our threading-based approach enables us to extend coverage of structure-based studies further than that possible by homology models (see the 'MAPK interactome validation' section). As a result, the predicted structures are more reliable and provide a sound basis for mechanistic hypotheses. We provide an anecdotal example by analyzing the distribution of annotated missense SNPs in our predicted models. In agreement with a recent study [63], we show that such mutations are enriched at the interfaces. Furthermore, detailed analysis of phosphorylation sites enables us to propose a crosstalk mechanism involving an atypical kinase, MAPK6. Predictions made by our model for the potential interactors of MAPK6 provide the basis for further exploration of the role of this relatively less-studied kinase.

Conventional homology-based methods such as interPrets [44], IBIS [73] and PRISM [45] perform well when a similar template is found in the PDB. Threading based-methods provide predictions even when such conventional methods cannot find a suitable template. Furthermore, as we show in this paper, accuracy achieved by our threading-based method is the best amongst current structure-based methods. Coev2Net acts as a complement to conventional homology methods whenever a clear template for prediction is not available and expands threading methods by incorporating coevolution of protein interfaces. However, performance of threading-based techniques has been shown to decline when the query sequences are distantly related to the template (sequence identities < 15 to 20%) [49,65]. While we currently use RAPTOR for identifying the putative interface, we hope to further push this limit by integrating new threading programs like RAPTORX [90] and iWRAP [49] into Coev2Net. While we encode our interface profile as a spanning-tree based graphical model, we believe this is a simplistic approximation of the reality. More complicated graphs could potentially be required for particular families of interacting proteins. Finally, we note that transient interactions are notoriously difficult to predict using structure-based interactions. Our validation using a technique (LUMIER) that can detect even transient interactions provides some confidence in predictions of transient interactions by Coev2Net.

## Materials and methods

### Coev2Net algorithm

The Coev2Net algorithm can be roughly divided into three distinct stages: 1) identification of the putative binding interface; 2) evaluation of the compatibility of the interface with an interface co-evolution-based model (see 'Construction of the interface profile through simulated co-evolution' below); and 3) evaluation of the confidence score for the interaction.

#### Identification of the putative interface

The two query sequences are each threaded against a complex template library to search for the best template. We use a top-performing threader program 'RAPTOR' [69,90] to look for the best template match. Given a set of potential template matches, the best match is selected based on the z-score of the alignment. In order to evaluate the putative interface implied by the alignment, we calculate its compatibility with respect to the co-evolutionary profile for that interface.

#### Evaluating the interface

The predicted interface is evaluated by computing the log-likelihood of the interface residues with respect to the interface profiles described below - a PGM for interacting pairs ('positive') and another graphical model representing background correlations ('negative'). A high log-likelihood with respect to the 'positive' PGM implies that the protein sequences show co-evolution at the interface, compatible with the model, and are hence likely to interact.

#### Computing confidence score

Once we have the compatibility scores for the predicted interface, we use these as features to predict our confidence in the interaction. A logistic-regression classifier is trained on a high-confidence network, and is used to predict our confidence score for the interaction, which is the output of the classifier. Both alignment features (from stage 1: Identification of interface) and interface features (from stage 2: Evaluating the interface) are used as features in the classifier. If *p *is the probability of interaction (or our confidence score), then:

log p1-p=α+β1TX1+β2TX2+βiYi+β+TL++β-TL-

where *X*_i _are the alignment features for each protein in the interacting pair (these include sequence scores, secondary structure scores and protein lengths); *Y*_i _is the size of the interface; *L*_+ _is the log-likelihood score of the predicted interface with respect to the positive tree, and *L*_- _the log-likelihood score of the predicted interface with respect to the negative tree. The α, β1, β2, βi, β+ and β- are coefficients of the classifier.

### Construction of the interface profile through simulated co-evolution

To construct an interface profile for a SCOPPI family, which consists of a family of protein complexes; we exploit the biological intuition that interacting proteins exhibit co-evolution at the interface. This co-evolution has been detected even in residues within 10 to 12 Ångströms at the interface [62,64,91-94]. In Coev2Net, the interface profile is a probabilistic graphical model (PGM), pre-computed for each SCOPPI family, and encodes the most significant pattern of interface correlations exhibited by the interacting members of the SCOPPI family. This model is computed by formulating interface co-evolution as a high-dimensional sampling problem (see Additional file 1 for further details). The three main steps in this simulation are seeding the co-evolution, simulating co-evolution for an interface and learning the PGM.

#### Seeding the co-evolution

We start the simulation from known complexes within a SCOPPI family. We first align the interfaces using a contact map alignment program, CMAPi [95]. CMAPi employs a contact map representation to efficiently align multiple interfaces and thereby improves alignments as compared to other sequence and structure-based techniques. The simulation is performed on each aligned interface.

#### Simulating co-evolution for an interface

For each pair of aligned seed sequences (full proteins forming the complex), additional sequences are constructed via random mutations according to a probability distribution (Figure S1 in Additional file 1) based on paired positions within interfaces of complexes. To perform a mutation at a contact, we first randomly fix one amino acid in the contact, and sample the contacting amino acid from a distribution conditioned on the fixed amino acid (see Figure S2 in Additional file 1 for a schematic). The new contact thus has one amino acid as before, and the contacting amino acid mutated according to a conditional probability distribution. Each contact is treated independently, with 5% of the interface contacts mutated at each step. For non-contacting residues, mutations are performed independently in the two proteins according to the BLOSUM62 matrix. Again, 5% of the non-contacting residues are mutated in one step. The percentage of mutations to carry out in one step (that is, 5%) was chosen based on previous studies on simulated evolution for remote homolog detection [96].

The new sequences are first aligned to the hidden Markov models (HMMs) representing the corresponding protein families, and the alignment scores computed. They are then accepted or rejected in a stochastic manner, based on their joint fitness score. The mutation and stochastic selection of interacting sequences can be viewed as a Markov chain Monte Carlo (MCMC) algorithm [97] for a high-dimensional sampling problem - we rigorously prove this correspondence in the supplemental methods in Additional file 1.

#### Learning the PGM

Once we have sufficient sequences (that is, after the MCMC converges), we encode the pairwise correlations observed in these 'interacting' sequences using a PGM. Our motivations for introducing a PGM are twofold: 1) analogous to a sequence profile, a PGM is a 'profile' that can be used to score predicted interfaces; and 2) to explicitly capture long-distance correlations (non-contact-based) at or near the interface residues. We select 1,000 interacting sequences per training complex as our interacting set (to avoid large sample-sample fluctuations, we select close to 2,500 sequences for SCOPPI families having only one training complex). To model the correlations between residues of these interacting proteins, we use the Sanghavi-Tan-Willsky algorithm [98] to construct two trees - one for the simulated interacting proteins ('positive') and one for background correlations ('negative'). These two trees are our interface profiles for the particular SCOPPI family and can be pre-computed before making any predictions. We restricted ourselves to spanning trees for ease of learning and inference. In fact, other inference methods, such as belief propagation, would work on a loopy graph (that is, the loopy network of contacts at the interface) but their behavior is not easy to control and very sensitive to the initialization. Note that our profiles of the interface residues are different from the HMM ones since our interface profiles are purposely computed from only interacting sequences; the HMM is constructed from independent sequences that do not necessarily interact.

### Evaluation of the classifiers

The individual methods were evaluated based on their ability to correctly predict true-positives and true-negatives. To do this, we plot receiver operator characteristic (ROC) curves for each method. In our ROC curves, sensitivity is defined as True-positives/(True-positives + False-negatives) and specificity is defined as True-negatives/(True-negatives + False-positives). For a high-confidence true-positive and true-negative dataset, we perform five-fold cross-validation (CV) tests for each method (Coev2Net, Struct2Net and Baseline), and plot the average sensitivities (at particular specificities) for these five runs. For Coev2Net, we run the MCMC sampling 5 times, and average the performance across these 25 curves (5 MCMC × 5 CV). To compare against interPrets, we used a cutoff on the z-score computed by the algorithm to classify a prediction as positive or negative. Since there is no training required here, there was no need for a cross-validation. For the computationally intensive IBIS [73], we compared our predictions on the ten pairs validated using the LUMIER assay.

## Abbreviations

HMM: hidden Markov model; HTP: high-throughput; MAPK: mitogen-activated protein kinase; MCMC: Markov chain Monte Carlo; PGM: probabilistic graphical model; PPI: protein-protein interaction; ROC: receiver operator characteristic; SNP: single nucleotide polymorphism; Y2H: yeast-2-hybrid.

## Competing interests

The authors declare that they have no competing interests.

## Authors' contributions

RH, JB, and BB conceived and designed the study. RH designed and implemented the algorithm; JP and RH provided the proof. JP, JB and BB helped in designing the algorithm and interpretating the results. JP, AV, JX and US provided tools, protocols and reagents. AV and US did the LUMIER experiments. AV, NP and US provided feedback on the manuscript and suggested applications for the algorithm. RH, JP, JB and BB wrote the manuscript. All authors read and approved the final manuscript.

## Supplementary Material

Additional file 1 **Supplementary methods on the algorithm, results on benchmarking and comparison with other methods**. Click here for file
