# Supplementary material for: A computational framework for boosting confidence in high-throughput protein-protein interaction datasets
Source: Genome Biol. 2012 Aug 31;13(8):R76. doi: 10.1186/gb-2012-13-8-r76 (PMC4053744; doi:10.1186/gb-2012-13-8-r76)
Supplement: Additional file 1 — Supplementary methods on the algorithm, results on benchmarking and comparison with other methods. [file gb-2012-13-8-r76-S1.PDF]

Supplement: A computational framework for boosting confidence in high-throughput protein-protein interaction datasets

Raghavendra Hosur[1], Jian Peng[1,2], Arunachalam Vinayagam[4], Ulrich Stelzl[7],Jinbo Xu[2],Norbert Perrimon[4,6], Jadwiga Bienkowska[3,8], Bonnie Berger[1,5,8]

[1] Computer Science and Artificial Intelligence Laboratory, MIT, Cambridge, MA
[2] Toyota Technological Institute, Chicago, IL
[3] Biogen Idec, Cambridge, MA
[4] Department of Genetics, Harvard Medical School, Boston, MA
[5] Department of Mathematics, MIT, Cambridge, MA
[6] Howard Hughes Medical Institute, Harvard Medical School, Boston, MA
[7] Max Planck Institute for Molecular Genetics, Berlin, Germany
[8] Corresponding Author, email: bab@mit.edu, jbienkowska@gmail.com

1 The Coev2Net algorithm

We developed Coev2Net (**Fig 2**), an algorithm that exploits conservation of residues in and around the interface to predict protein-protein interactions. Coev2Net consists of four stages: 1) seeding the co-evolution, 2) simulating co-evolution, 3) construction of a probabilistic graph, and 4) prediction.

Stage 1: Seeding the co-evolution. To overcome sampling issues, we start from regions in the sequence space that we know are in high-probability interaction regions. Therefore, we seed the co-evolution with data from known complexes. For a given SCOPPI family, the set of training complexes are aligned using the alignment program CMAPi [3]. CMAPi employs a contact map representation to efficiently align multiple interfaces and thereby improve alignments as compared to other sequence and structure based techniques [3]. A contact map is a binary matrix representation of the residue-residue interactions between two proteins. If the distance between any two heavy atoms of the two residues is less than 4.5 Å, the corresponding entry in the contact map is 1, and 0 otherwise. In the following steps, the aligned interface sequences are used for the initialization (seed) of co-evolution.

Stage 2: Simulating co-evolution. Similar to the natural process of evolution, our simulation has a mutation and a selection step for the evolved sequences.

Mutation. For each pair of aligned seed sequences (full proteins forming the complex), additional sequences are constructed via random mutations according to a probability distribution based on paired positions within interfaces of complexes (**Figs S1**). To perform a mutation at a contact, we first randomly fix one

amino acid in the contact, and sample the contacting amino acid from a distribution conditioned on the fixed amino acid (See Fig S2a for a schematic). The new contact thus has one amino acid as before, and the contacting amino acid mutated according to a conditional probability distribution (from Fig S1). Each contact is treated independently, with 5% of the interface contacts mutated at each step. For non-contacting residues mutations are performed independently in the two proteins according to the BLOSUM62 matrix. Again, 5% of the non-contacting residues are mutated in one step (Fig S2b). The percentage of mutations to carry out in one step (i.e. 5%) was decided based on previous studies on simulated evolution for remote homolog detection [2].

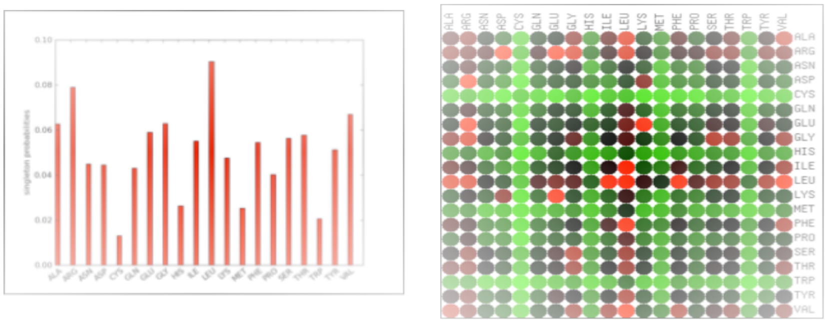

Fig. S1: Singleton and pairwise probabilities at the interface computed from a database of non-redundant complexes. In the rightmost figure, red indicates high probability values, green indicates low and black, the mid-range value

Selection. The new sequences are first aligned to the HMMs representing the corresponding families [6], and the alignment scores computed. They are then accepted or rejected in a stochastic manner, based on their joint fitness score (Fig S2b). If E^1 and E^2 are the (negative) alignment scores for the two evolved sequences w.r.t the HMMs, then the following function α is computed and used to select new sequences:

$$\begin{aligned} \alpha &= (P^{new} \Pi_j p_j^{old})/(P^{old} \Pi_j p_j^{new}) \\ P &\propto exp(-E^1 - E^2) \\ p_j &= q_{uniprot},\ j \text{ is not an interface position} \\ &= q,\ \text{otherwise} \end{aligned} \tag{1}$$

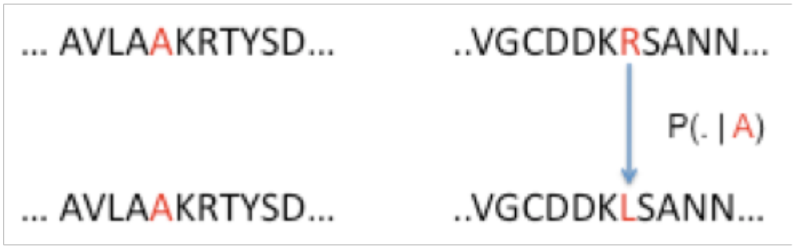

(a)

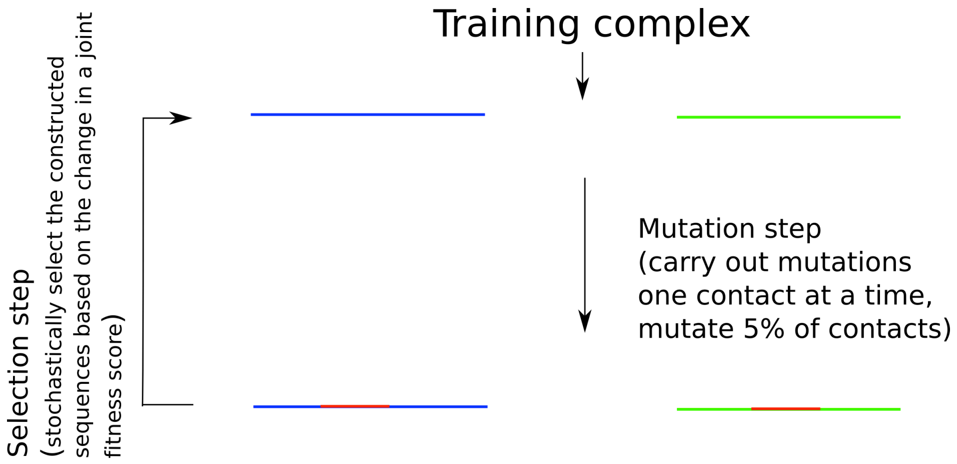

(b)

Fig. S2: a) Mutating a single contact at the interface. The red color indicates the contact selected to be mutated. The blue arrow represents the mutation step, with the corresponding distribution from which the mutated amino acid is drawn indicated. b) For each interface (complex) in the training set, 5 % of the contacts are mutated as in a and 5% on the non-contacting residues are mutated using a BLOSUM62 matrix. The new sequences are stochastically accepted or rejected by calculating the change in a joint fitness score (α)

where $q_{uniprot}$ is the amino-acid distribution in Uniprot; q the amino-acid distribution at the interface from a selected non-redundant set of complexes (**Fig S1**); and α the probability of the mutations at the interface being accepted. If $\alpha > 1$, the new sequences are accepted automatically. However, to incorporate diversity into the evolved sequences, we also accept sequences with a certain probability even if this ratio is low. A random number is drawn uniformly from [0,1], and the new sequences are accepted if this number is less than α. Intuitively, α represents how likely it is the sequences (interface) belong to the co-evolving families, as compared to a model that considers all positions independent. We show that simulated co-evolution, viewed through the lens of a high-dimensional sampling problem, leads to the same co-evolution and selection step (see proof below). Along the course of the simulation, we monitor the sum of the entropies of all the sequence positions, and only retain sequences at an interval of 10 iterations after this value converges. These sequences are non-redundant representatives of their respective families, with the added feature that they are assumed to be interacting.

Stage 3: Interface profile (PGM). A probabilistic graphical model (PGM) is then constructed for a particular SCOPPI family, based on the observed correlations at the interface in sequences simulated by co-evolution (stage 2). Once the MCMC has converged, we sample 1000 interacting sequence pairs per training complex as our interacting set. To model the correlations between residues of the interacting proteins, we use the Sanghavi-Tan-Willsky algorithm [4] to construct two trees– one for the simulated interacting proteins and one for background correlations. The trees are the maximum-likelihood estimate over tree-like graphs of the generating distribution for the simulated sequences. They are computed by solving a maximum-weight spanning tree problem on a graph whose nodes are the residues and edges are weighted by the mutual information between the two residue positions (nodes). The mutual information between two residue positions is calculated from the corresponding empirical pairwise and singleton distributions in the set of simulated sequences. For more details, we refer the reader to Sanghvi, Tan and Willsky [4]. The maximum-weight spanning tree problem within STW is solved using NetworkXs implementation of Kruskals algorithm [1]. Our choice of a tree graphical model is mainly due to the computational issues; trees are easy for both learning and inference. The PGMs are pre-computed and used for prediction.

Stage 4: Classifier. In the final stage, we build a classifier to predict protein-protein interactions using the probabilistic graphical model. For a given pair of proteins for which we need to predict interaction, we first predict the interface through threading to a suitable template (see main text). For this predicted interface, we evaluate the interface using the PGMs corresponding to the template. The interface is evaluated by calculating the log-likelihood of the predicted interface residues w.r.t the PGM. We split the tree log-likelihood scores into edge (i.e residue-residue propensity) and node (i.e. single residue propensity) contributions, and train a logistic-regression classifier using these scores. Additionally, we include sequence lengths, the alignment features from the threading and size

of the trees as features in the classifier (see main text). We further use cross-validation to train the classifiers and avoid overfitting.

1.1 Proof of equivalence of simulated co-evolution and high-dimensional sampling

Our procedure for simulated co-evolution involving the mutation/selection steps is equivalent to a high-dimensional sampling problem. We can model evolution as nature sampling from a complicated distribution that describes the interacting sequences in the two families (of the proteins). The distribution, which can be modeled as a graph, has the two HMMs, one for each family, and edges at the interface to couple the two HMMs together. In general, calculating the partition function and profiles (or marginals) is computationally intractable [7]; therefore we use a Markov Chain Monte Carlo (MCMC) technique to draw sample sequences from this distribution. If E^1 and E^2 are the (negative) alignment scores for the two evolved sequences w.r.t the HMMs, then we assume the form of this distribution to be:

$$\begin{aligned} P^{eq} &\propto exp(-E^1 - E^2 - E^{int}) \\ E^{int} &= - \sum_{interface} log(Q(a,b)/q(a)q(b)) \end{aligned} \tag{2}$$

where the interface energy term E^{int} is obtained by summing over all contacts (a, b). $Q(q)$ is the pairwise (singleton) distribution shown in **Fig S1**. Let $X_i^1, X_j^2, i = 1..n, j = 1..m$ be the amino acids at the interface ($< 10\mathring{A}$) of the two interacting proteins (complex) that are in the contact map constructed by CMAPi. At each iteration of the MCMC, the goal is to construct $X_i^{1,new}, X_j^{2,new}$ from $X_i^{1,old}, X_j^{2,old}$ by mutating a fraction of the residues. We treat each contact independently, so we can look at the mutation for a single contact. For each contact (i, j) at the interface, we first randomly select a protein from the pair and fix the corresponding amino acid in the contact. Let that protein be 1, say. The contacting amino acid in protein 2 (at position j) is then chosen from the following probability distribution (see **Fig S2**):

$$\begin{aligned} X_j^{2,new} &\sim Q(.|X_i^{1,new}) \\ X_i^{1,new} &= X_i^{1,old} \end{aligned} \tag{3}$$

where the conditional probabilities are computed from the distributions in **Fig S1**. For non-interface residues, the BLOSUM62 matrix is used (by computing the conditional probabilities) to mutate residues independently in the two proteins. The new sequences are then aligned to the HMMs representing their families and are accepted or rejected using a Metropolis-Hastings criterion based on their alignment scores and the interface energy E^{int}.

Metropolis-Hastings criterion Since we treat each contact independently while sampling, let us assume for the sake of simplicity that there is only one contact (a, b). In the simulated sequences, this is evolved to (a', b). Because we simulate co-evolution of the contact one residue at a time, the ratio of transition probabilities will be (old $\rightarrow$ new over new $\rightarrow$ old):

$$J^{int} = Q(a|b)/Q(a'|b) = Q(a,b)/Q(a',b) \tag{4}$$

where Q is the pairwise distribution shown in **Fig S1**. For the mutation of non-interface residues, since the two partners are mutated independently, the ratio of transition probabilities will just be the product across all non-interface positions:

$$\begin{aligned} J^{non-int} &= \Pi q_{uniprot}(x^{old}|x^{new})/\Pi q_{uniprot}(x^{new}|x^{old}) \\ &= \Pi q_{uniprot}(x^{old})/\Pi q_{uniprot}(x^{new}) \end{aligned} \tag{5}$$

where $q_{uniprot}$ is the Uniprot distribution. The Metropolis-Hastings criterion can then be written as:

$$\begin{aligned} \alpha &= P^{eq}(X^{new}) * J^{int} * J^{non-int}/P^{eq}(X^{old}) \\ P^{eq}(X^{new}) &= P^{new} * Q(a',b)/(q(a')q(b)) \\ P^{eq}(X^{old}) &= P^{old} * Q(a,b)/(q(a)q(b)) \\ P &\propto exp(-E^1 - E^2) \end{aligned} \tag{6}$$

Note that the pairwise probability terms, $Q(a, b)$ and $Q(a', b)$, in $P^{eq}(X^{new}) * J^{int}/P^{eq}(X^{old})$ cancel each other, leaving only the product of singleton probabilities. Therefore:

$$\begin{aligned} &\alpha = (P^{new}\Pi_j p_j^{old})/(P^{old}\Pi_j p_j^{new}) \\ &P \propto exp(-E^1 - E^2) \\ &p_j = q_{uniprot}, \ j \text{ is not an interface position} \\ &\quad = q, \ \text{otherwise} \end{aligned} \tag{7}$$

where recall that $q_{uniprot}$ is the amino-acid distribution in Uniprot; q the amino-acid distribution at the interface from a selected non-redundant set of complexes (**Fig S1**). This is exactly our fitness score used to select the co-evolved interfaces in the Selection step. QED

Note that this MCMC procedure allows us to efficiently compute any pairwise correlations, even those that are not contact based; a feature not possible without our sampling-based procedure.

2 Datasets

All crystal structures were obtained from the Protein Data Bank (PDB). Singleton and pairwise amino-acid probabilities at the interface were calculated

from a 50% non-redundant set of complexes downloaded from the 3DComplex database http://supfam.mrc-lmb.cam.ac.uk/elevy/3dcomplex/Home.cgi. Here, two residues were assumed to be interacting if any heavy atom in one residue on one protein was at a distance of less than 5 from any heavy atom on the other residue in the partner protein. The calculated singleton and pairwise probabilities calculated are shown in the Fig S1. As one would expect, hydrophobic residues (A, V, L) are highly represented at the interface, whereas cysteine has the lowest propensity. Interestingly, Arg, Gly and Glu show up with a high propensity as well, indicating a preference for ionic and H-bond interactions at interfaces. This is in contrast to the general composition in globular proteins, where Arg is less frequent than Ala, Glu, and Gly is found at a much lower frequency .

All the MAPK PPI data was taken from Bandyopadhyay et al. (2010) and Vinayagam et al. (2011). The negative dataset used in evaluation of the classifier (PDB-negative) was downloaded from the negatome database http://mips.helmholtz-muenchen.de/proj/ppi/negatome/. In these datasets, only the sequences that could be aligned to templates belonging to families for which we could apply the simulated evolution protocol were considered. Sequences that had a z-score less than 5 for their alignment were discarded and such alignments were deemed not confident enough to give an accurate inference. In the Bandyopadhyay set, we could get predictions for 461 interactions; in the Vinu set, 1025 interactions, and in the negatome (PDB-negative set), 330 non-interactors. The Bandyopadhyay set was further divided into a 173 Core set of interactions, defined by the authors, and the rest as non-core.

3 Results

3.1 Coev2Net benchmarking

To carry out the benchmarking for Coev2Net, we evaluate performance on known complexes in SCOPPI, a database of structural interfaces. SCOPPI records homomeric structural complexes as well and defines biologically relevant interfaces using number of contacts and buried surface area. We have used the definition of biounits as implemented in SCOPPI to deal with interfaces due to crystallographic symmetry. In our predictions, we have not used homomeric complexes for threading.

Cross-validation on SCOPPI. For each family in SCOPPI having three or more non-redundant complexes ($< 50\%$ sequence identity), we randomly select one as a Test Set and the remaining complexes as the Training Set. RAPTOR [8] is used to align the test sequences to the training templates, and the best alignment (based on RAPTOR's z score) selected for evaluation. Because of limited datasets ($\sim$ 45 families that meet our criterion of non-redundancy in SCOPPI and $\sim$ 300 negative pairs from the manually curated PDB-negative set (see Datasets)) [5], we use a 5-fold cross-validation to train and test the classifier.

Limited complex families. Additionally, for SCOPPI families that have only two non-redundant complexes, Coev2Net gives similar results (**Fig S3**). To test

on these families, one complex (of the two) was chosen randomly, and the correlation graph computed as before, except for the multiple interface alignment stage. The classifier trained on multiple complex families was used to compute the probability of interaction of the test complex. As can be seen in **Fig S3**, the algorithm is able to successfully use relevant correlations, even in the absence of multiple complexes for a given family, to help identify conserved structural features. Note that iWRAP cannot handle such families as it cannot build interface profiles due to the limited number of complexes.

3.2 Abundance of SNPs

To compute association between PolyPhen annotations ('benign' and 'damaging') and our prediction of the SNP's location, we calculated the p-value using a 2x2 contingency table. Similarly to calculate association between SNPs and the location, we computed the p-value using a 2x2 contingency table with one grouping as total number of interface/non-interface residues and the other grouping as the occurrence/non-occurence of a SNP at that location. To verify abundance, we first normalized the occurrence of a SNP at a site by the number of such sites in the protein (a site is either an interface or a non-interface), and then performed a mann-whitney (paired) test to compute the p-value for the difference between the mean of the two densities (for the two types of sites).

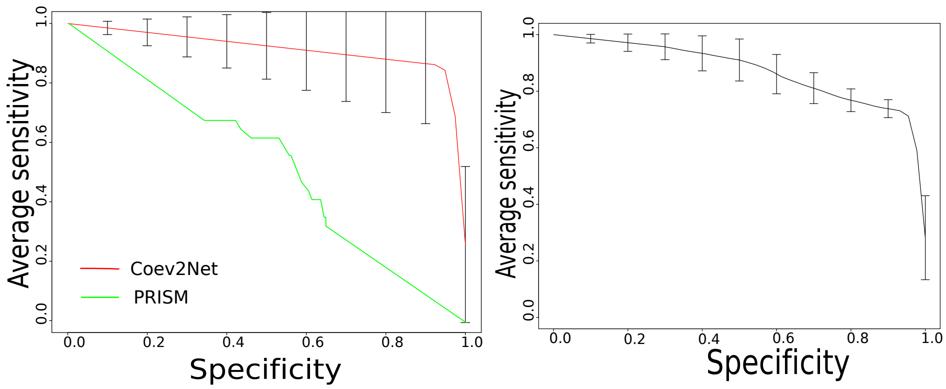

Fig. S3: Cross-validation results on SCOPPI. (left) Results on SCOPPI families having 3 or more complexes. (right) Results on SCOPPI families having only 2 complexes (1 training and 1 test)

References

1. http://networkx.lanl.gov/

Interactor A	Interactor B	SeqID
GNB2	GSK3B	0.27
RBPJ	GSK3B	0.32
**MAP3K7IP1**	**MAPK14**	**0.28**
**IDH3B**	**MAPK6**	**0.30**
**MAP3K7IP1**	**CASP6**	**0.23**
MAP3K7IP1	MAPKAPK5	0.28
**MAPK11**	**CPNE6**	**0.33**
**MAPK11**	**MAPK14**	**0.32**
**MAPK6**	**ANAPC5**	**0.33**
**MAPK6**	**CALR**	**0.30**
MAPK6	PRKAR1A	0.30
MAPK6	PSAT1	0.31
MAPK8IP2	NDUFS6	0.24
MAPK6	PTPMT1	0.28
**RBPJ**	**GADD45A**	**0.20**
RPS6KA6	C14orf1	0.25
**RPS6KA6**	**MAPK3**	**0.34**
SMARCB1	RPS6KA5	0.29
**UNC119**	**RPS6KA5**	**0.23**

Table 1: Average sequence identities between the sequences and templates used by Coev2Net to make a prediction. The 10 pairs experimentally validated using LUMIER are shown in bold.

2. Daniels, N., Hosur, R., Berger, B., Cowen, L.: Smurflite: combining simplified markov random fields with simulated evolution improves remote homology detection for beta-structural proteins into the twilight zone. Bioinformatics (2012), doi: 10.1093/bioinformatics/bts110
3. Pulim, V., Bienkowska, J., Berger, B.: Optimal contact map alignment of protein-protein interfaces. Bioinformatics 24, 2324–2328 (2008)
4. Sanghvi, S., Tan, V., Willsky, A.: Learning graphical models for hypothesis testing. Statistical Signal Processing Workshop (SSP) (2007)
5. Smialowski, P., Pagel, P., Wong, P., Brauner, B., Dunger, I., Fobo, G., Frishman, G., Montrone, C., Rattei, T., Frishman, D., Ruepp, A.: The negatome database: a reference set of non-interacting protein pairs. Nucleic Acids Research D38, D540–544 (2010)
6. Sōding, J.: Protein homology detection by hmm-hmm comparison. Bioinformatics 21, 951–960 (2005)
7. Sorin, I.: Statistical mechanics, three-dimensionality and np-completeness: I. universality of intractability of the partition functions of the ising model across non-planar lattices. Proceeding of the 32nd ACM symposium on the theory of computing (STOC00) pp. 87–96 (2000)
8. Xu, J., Li, M., Kim, D., Xu, Y.: RAPTOR: Optimal protein threading by linear programming. J Bioinform Comput Biol 1, 95–117 (2003)
